# The prevalence of metabolic syndrome among university students in Wasit, Iraq

**DOI:** 10.15537/smj.2022.43.11.20220558

**Published:** 2022-11

**Authors:** Alaa H. Zamil, Seenaa S. Amin

**Affiliations:** *From the Department of Clinical Laboratory Sciences, College of Pharmacy, University of Baghdad, Baghdad, Iraq.*

**Keywords:** metabolic syndrome, prevalence, university student, risk factors, lifestyle

## Abstract

**Objectives:**

To determine the prevalence of the metabolic syndrome (MetS) and its related risk factors in a group of healthy subjects.

**Methods:**

This cross-sectional analytic investigation used a convenient sample of 300 apparently healthy university students from Wasit, Iraq, between October 2021 and February 2022. The data was collected using a structured direct interview with a self-administered questionnaire. Anthropometric measurements (waist circumference [WC], body mass index [BMI], height, weight, and the blood pressure), total cholesterol level, triglyceride level, high-density lipoprotein (HDL-C), and fasting blood glucose (FBG) were all measured. IDF/AHA/NHLBI criteria were used to diagnose metabolic syndrome.

**Results:**

Overall, 41.3% of students had MetS, with female (66.9%) students having the highest frequency. The most common component of MetS was elevated FBG (98.3%), followed by increased WC (87.9%), and finally a low HDL-C level (85.4%). The following factors were found to be predisposing to MetS: being female (OR=2.32), over the age of 20 (OR=1.96), obese (OR=9.46), high consumption of fast food (OR=2.35), and physically inactive.

**Conclusion:**

Metabolic syndrome prevalence and defining criteria are significantly high among Iraqi university students. Fasting blood glucose was the most common component followed by increased WC. The significant risk factors for MetS were older age, females, high BMI (≥25), low physical-activity, and eating of fast foods, and can remedy the risk if the components of the disease are reported at a younger age.

**M**etabolic syndrome (MetS) prevalence has been rising steadily over the past few decades all over the world. This condition, which is now considered a major problem in both public health and clinical practice, is approaching epidemic levels.^1^ Metabolic syndrome has been linked to an increased risk of diabetes as well as both atherosclerotic and non-atherosclerotic cardiovascular disease (CVDs). The hallmarks of MetS involve central obesity, hyperglycemia, hypertention, and abnormal lipid profiles.^2^ The leading factor in patient death and morbidity, particularly in diabetes patients, is CVDs.^3^ As reviewed previously several organizations have attempted to create MetS diagnostic criteria. The World Health Organization (WHO) made the first attempt in 1998, defining MetS as a syndrome categorized by insulin resistance and the presence of insulin resistance was an essential component of the syndrome, with a minimum 2 from following criteria: raised triglycerides, low high density lipoprotein (HDL-C) levels, raised blood pressure, obesity, and microalbuminuria. In 2001 The National Cholesterol Education Program (NCEP) published a new group of criteria that included fasting blood glucose (FBG), blood pressure, dyslipidemia, and waist circumference (WC). While in 2005 the International Diabetes Federation (IDF) added central obesity as a requirement for MetS diagnosis, focusing on waist circumference as a simple screening tool.^4^ The IDF and the American Heart Association/National Heart, Lung, and Blood Institute (AHA/NHLBI) carried out a research in 2009 to determine whether or not 3 of the 5 criteria for MetS were present, and it was relied upon in the present research.^5^

The global prevalence of MetS in young adults ranged from 5% to 7%, whereas the results of the National Health and Nutrition Examination Survey (NHANES) showed that the prevalence of MetS rose dramatically among people aged 20-39 years old in the United States (from 16.2% to 21.3%), and among Asian race individuals (from 19.9% to 26.2%).^6,7^ The presence of one component of MetS increases the chance of acquiring MetS in the future and likely represents a significant lifetime burden of CVD risk. This increases the risk of having CVD later in life.

According to a systematic study that was carried out by Campo-Arias et al,^8^ there is a large amount of heterogeneity of MetS prevalence in college students. This prevalence ranges from 0% to 19.2% according to NCEP-ATP III definition.^8^ This varied may be due to influenced by their new surroundings and social influences, which can lead to hazardous behaviors. Unhealthy diets and a low levels of physical activity have been the most common harmful behaviors. Any of these factors can lead to overweight and an increase in MetS prevalence and associated symptoms.^9^

According to a previously reported analysis, MetS prevalence and its components is influenced by a variety of factors, including genetic background, levels of physical activity, diet, diabetic family history, smoking, and educational attainment.^1,4^

Early detection of MetS components may result in focused therapies that stop the syndrome from developing and, as a result, minimizes the risk of CVD later in life. There are few published statistics on the prevalence of MetS among young adults in Iraq. Therefore, this study aimed to determine MetS prevalence among Iraqi university students and recognize potential risk factors for MetS prevention and management in young adults.

## Methods

This cross-sectional study participants were selected from Wasit University, Technical Institute, and Kut University College, Wasit, Iraq, between October 2021 and February 2022. A convenient sample of 300 students for this study of 18-25 years from mixed colleges and stages were chosen. The inclusion criteria were adults (18-25 years old), both genders, and have no chronic disease. The exclusion criteria were CVD patients, diabetic or hypertensive patients, autoimmune disease patients, pregnant and breastfeeding women, and patients with kidney and liver problems. A verbal agreement was obtain from all participants after being informed on the study’s purpose and the expected benefits. The research was carried out in accordance with the Helsinki Declaration.

A formalized direct meeting with questionnaires managed by the interviewer was used. The questionnaire included requests on demographics, sedentary lifestyle, physical activity, and dietary habits. The amount of fruit and vegetables eaten daily was determined by tallying up the total number of servings of fruit and vegetables consumed on a daily basis over the course of a typical week. Insufficient consumption of fruit and vegetables was defined as having fewer than 5 servings on a daily basis. The question, “On average, how many meals per week do you consume that were not made at home?” was used to determine how often respondents ate meals away from home, specifically fast food. Daily eating of fast food (≥1-3 times/week) considered eating. The smoking status were divided into 2 groups regular active smokers (smoking at least 10 cigarette/week) and non-smokers, and type of smoking classified to cigarette, Argela, and both.

It was suggested that engaging in physical exercise of a moderate level for more than 150 minutes a week 10 should be considered sufficient to keep one in a decent condition of health. This was the dividing line that our research used to separate participants who engaged in regular physical activity from those who did not participate in such activity.

Following a 5-minute break time, consented participants were provided a verified self-questionnaire. Once completed, anthropometric data of BMI and waist circumference were taken by a trained nurse.

Weight, length, and waist measurement were all considered. A portable stadiometer (Seca, Germany) was used to measure height, a portable electronic scale (Germany) was used to measure weight, and a non-stretchable tape for the measurement of waist circumference, when exhaling, WC was measured at the midway between the top of the hip bone and the lowest rib. During the weighing, participants were asked to dress light clothing and refrain from wearing shoes. Weight was recorded in kilograms and height was recorded in meters. Body mass index (BMI) (kg/m^2^) was calculated using weight and height. Underweight (BMI: <18.5), normal weight (BMI: 18.5-24.9), overweight (BMI: >25), and obese (BMI: >30) were used to categorize participants.^10^

The measurement of systolic and diastolic blood pressures (SBP and DBP) after a 15-minute rest by using a standardized mercury sphygmomanometer (MDF, USA), and it was measured twice at least a half-hour gap, and the participant’s BP was calculated using the average of 2 readings.

The biochemical tests listed below were performed on blood samples that were drawn in the morning after a 12-hour fast. An enzymatic colorimetric method with readymade kits was used to measure fasting serum glucose (FSG), triglyceride (TG), serum cholesterol, and HDL: Glucose MR (fasting) kit (LINEAR CHEMICALS, SPAIN), Triglycerides MR kit (LINEAR CHEMICALS, SPAIN), HDL- cholesterol kit (LINEAR CHEMICALS, SPAIN), and Cholesterol CHOD PAP kit (Biolabo SAS, France).

According to the definition IDF/AHA/NHLBI criteria, participants had MetS when they had 3 or more of the following 5 criteria: i) A high FBG levels ≥100 mg/dL; ii) A high blood pressure reading (defined as a SBP ≥130 mmHg or a DBP ≥85 mmHg); iii) A low level of HDL-C (for male <40 mg/dL and female <50 mg/dL); iv) A high level of TG ≥150 mg/dL; and v) An increased waist circumference (male ≥37 inches (94 cm) and female ≥31.5 inches (80 cm).

### Statistical analysis

Data analysis was carried out using the SPSS version 25.0 software (IBM Corp., Armonk, NY, USA). The test of Shapiro-Wilk was used to evaluate whether the data were assumed to be normal. Continuous variable expressed as median (interquartile rang), and difference between groups were checked using Mann-Whitney tests. The categorical data are presented as percentage and frequency, and difference between groups were checked using Chi-square test. The association between the MetS group and a potential risk factor is measured using an odds ratio (OR) with 95% confidence intervals (CI). A *p*-value of <0.05 was deemed statistically significant.

## Results

A total of 300 students were included, 165 (55%) female and 135 (45%) male students. Their overall age was ranged (18-25) years. Over weight and obese students represented (31%, 7%) respectively of the research sample, 9.7% of them had regular physical activity. Most students were nonsmokers (244; 81.33%)(Table 1).

**Table 1:** - The distribution of anthropometric and lifestyle data among participants.

Characteristics	Total (n=300)		Female (n=165)		Male (n=135)		*P*-value
	n	%	n	%	n	%	
***Age (years)***							
18-21	160	53.3	96	58.2	64	47.4	0.063
22-25	140	46.7	69	41.8	71	52.6	
***Body mass index (Kg/m2)***							
Underweight (<18.5)	20	6.7	14	8.5	6	4.4	0.186
Normal (18.5-24.9)	166	55.3	93	56.3	72	53.3	
Overweight (25-29.9)	93	31	45	27.3	49	36.3	
Obese (≥30)	21	7.0	13	7.9	8	6.0	
***WC (inch)***							
<31.5 female	157	52.3	63	38.2	94	69.6	0.033*
<37 male							
≥31.5 female	143	47.7	102	61.8	41	30.4	
≥37 male							
***Systolic BP mmHg***							
<130	233	77.7	141	85.5	92	68.1	<0.001**
≥130	67	22.3	24	14.5	43	31.9	
***Diastolic:***							
<85	238	79.3	138	83.6	100	74.1	0.046*
≥85	62	20.7	27	16.4	35	25.9	
***Physical activity (min/week)***							
<150	271	90.3	160	97.0	111	82.0	<0.001**
≥150	29	9.7	5	3.0	24	18.0	
***Smoking***							
Yes	56	18.67	2	1.2	54	40.0	<0.001**
No	244	81.33	163	98.8	81	60.0	
***Smoking type***							
Cigarette	21	7.0	0	0	21	15.6	<0.001**
Argela	25	8.34	2	1.2	23	17.0	
Both	10	3.33	0	0	10	7.4	
***Fast food (times/week)***							
0	36	12.0	14	8.5	22	16.3	0.004*
1-3	146	48.7	94	57.0	52	38.5	
>3	118	39.3	57	34.5	61	45.2	
***Vegetable eating (times/week)***							
0	9	3.0	7	4.2	2	1.5	0.094
1-5	200	66.7	110	66.7	90	66.7	
>5	91	30.3	48	29.1	43	31.8	

WC: waist circumference, BP: blood pressure, *: significant difference (<0.05), **significant difference (<0.001)

Most of students had high levels of FBG (≥100 mg/dl) 90% male (94.1%) was significantly (*p*=0.034) higher than female (86.7%). Low levels of HDL-C were significantly higher in females than males (*p*<0.001) as shown in (Table 2).

**Table 2:** - Biochemical parameters statistics of participants.

Paraneters	Total (n=300)		Female (n=165)		Male (n=135)		*P* -value
	n	%	n	%	n	%	
FBG (mg/dl)							0.034*
<100	30	10	22	13.3	8	5.9	
≥00	270	90	143	86.7	127	94.1	
Triglycerides (mg/dl)							--
<150	300	100	165	100	135	100	
≥150	0	0	0	0	0	0	
HDL-C(mg/dl)							<0.001**
<50(female)			139	84.2			
<40 (male)	195	65			56	41.49	
≥50 (female)			26	15.8			
≥40 (male)	105	35			79	58.51	
Total cholesterol (mg/dl)							--
<200	300	100	165	100	135	100	
≥200	0	0	0	0	0	0	

FBG: fasting blood glucose, HDL-C: high density lipoprotein cholesterol, *significant (<0.05), **significant (<0.001)

**Figure 1:**
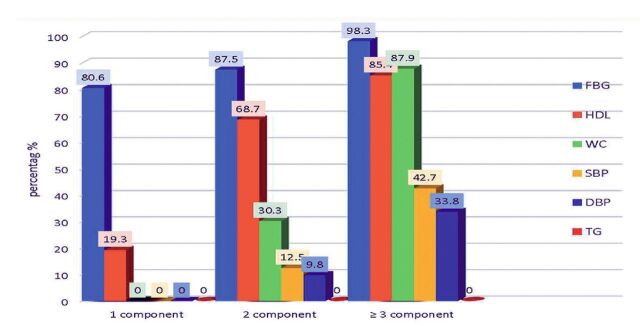
- Prevalence of metabolic syndrome criteria depending on the number of components. FBG: fasting blood glucose, HDL-C: high density lipoprotein cholesterol, WC: waist circumference, SBP: systolic blood pressure, DBP: diasolic blood pressure, TG: triglycerides

The prevalence of MetS using IDF/ AHA/NHLBI criteria was 41.3%, affecting 124 students. Sixty two (20.7%) students tested positive for one MetS criteria, 112 (37.3%) for 2 MetS criteria, 98 (32.7%) for 3 MetS criteria, and 26 (8.7%) students for 4 MetS criteria. There were no participants that were positive for 5 components (Table 3). Elevated FBG (98.3%) has been found to be the most prevalent MetS component, followed by increased WC (87.9%) and low HDL-C (85.4%), whereas elevated SBP was 42.7% and DBP was 33.8% (Figure 1).

**Table 3:** - The prevalence of MteS components among participants, according to IDF/ AHA/NHLBI criteria.

Component	Total (300)		Female (165)		Male (135)		*P*-value
	n	%	n	%	n	%	
0	2	0.6	0	0	2	1.5	0.117
1	62	20.7	15	9.1	47	34.8	<0.001**
2	112	37.3	67	40.6	45	33.3	0.196
3	98	32.7	67	40.6	31	23	0.001*
4	26	8.7	16	9.7	10	7.4	0.484

*significant (<0.05), **significant (<0.001)

Metabolic syndrome participants had significantly (*p*<0.05) higher weight, BMI, WC, SBP, DBP, FBG, TC, and reduced HDL C levels, and not significantly for TG levels (*p*=0.23) (Table 4).

**Table 4:** - The anthropometric measurements and biochemical parameter between groups of metabolic syndrome (MetS) and non-MetS.

Variables	Metabolic syndrome Median (interquartile range)		Z Test	*P*-value
	YES (n=124)	NO (n=176)		
Weight (kg)	71 (24.0)	62.5 (19.0)	6.05	<0.001**
Height (cm)	164.5 (13.0)	166 (17.0)	1.9	0.057
BMI (kg/m²)	26.55 (4.8)	22.22(4.1)	9.86	<0.001**
WC (inch)	37 (7.0)	31 (4.0)	9.66	<0.001**
SBP (mmHg)	125 (25.0)	115 (12.0)	5.54	<0.001**
DBP (mmHg)	82 (15.0)	75 (10.0)	6.1	<0.001**
Fasting blood glucose	113 (11.0)	110 (15.0)	2.7	0.006*
Total cholesterol (mg/dl)	167 (8.0)	165 (11.0)	2.64	0.008*
TG (mg/dl)	108 (5.0)	107 (5.0)	1.18	0.23
HDL-C (mg/dl)	39 (11.0)	42 (10.0)	2.5	0.01*

BMI: body mass index, WC: waist circumference, SBP: systolic blood pressure, DBP: diasolic blood pressure, HDL-C: high density lipoprotein cholesterol, TG: triglycerides

MetS was found to be significantly more common in female than male students (*p*<0.001) (OR [odds ratio]=2.321). The older participants were more at risk of developing MetS (*p*=0.004) (OR=1.963). As BMI increased, the prevalence of MetS rose, as it was high among participants with BMI ≥25 (66.9%) as compared to the 33.1% of participants who had BMI<25 (*p*<0.001). Moreover, low physical activity has been associated with a higher risk of the developing MetS among the participants (*p*<0.001). Regarding dietary habits, MetS was most common in individuals with increased consumption of fast food (51.6%) and low in individuals with increased consumption of vegetables and fruits (26.1%). The incidence of MetS was not significantly associated with smoking (*p*=0.57) according to small number of smokers in this study (Table 5).

**Table 5:** - Associations of metabolic syndrome with potential risk factors.

Risk factors	Metabolic syndrome		Chi-square test	*P*-value	Odds ratio	95% CI
	Yes n=(124)	No n=(176)				
***Age group (years)***						
18-21	54 (43.5)	106(60.2)	χ2 = 8.131	0.004*	1.96	1.23-3.13
22- 25	70 (56.5)	70(39.8)				
***Gender***						
Female	83 (66.9)	82(46.6)	χ2= 12.166	<0.001**	2.32	1.44-3.74
Males	41 (33.1)	94(53.4)				
***Smoking group***						
Smokers	25 (20.2)	31 (17.6)	χ2= 0.311	0.57	1.18	0.65-2.12
Non smokers	99(79.8)	145(82.4)				
***Type of smoking***						
Cigarettes+ both	11 (8.9)	20 (11.35)	χ2=3.49	0.06	3.18	0.92-10.9
Argela	14 (11.3)	11 (6.25)				
***BMI (Kg/m2)***						
<25	41 (33.1)	145(82.4)	χ2=75.113	<0.001**	9.46	5.52-16.23
≥25	83 (66.9)	31(17.6)				
***Physical activity (minute/week)***						
<150	124 (100)	147 (83.5)	χ2=22.6	<0.001**	0.54	0.48-0.60
≥150	0 (0)	29 (16.5)				
***Fast-food (times/week)***						
1-3	49 (39.5)	97 (55.1)	χ2=11.3	0.001*	2.35	1.42-3.86
>3	64 (51.6)	54 (30.7)				
***Vegetable eating (times/week)***						
1-5	88(73.9)	112(65.1)	χ2=2.55	0.11	0.65	0.39- 1.10
>5	31 (26.1)	60 (34.9)				

BMI: body mass index, CI: confidence interval

## Discussion

The fundamental result of this research was MetS has a prevalence of 41.3% (66.9% female and 33.1% male) in university-aged adults. This agreed with the findings for Egyptian university students: 16.7% MetS prevalence in 18-25 year old, which use the ATP-III guidelines.^11^ The risk of developing MetS prevalence in the Kingdom of Saudi Arabia among university students was 17.7%.^12^ While the study by Olfert et al^13^ reported that MetS prevalence among West Virginia University students was 15%.

Many studies in Iraq reported MetS prevalence in various populations. For example, Al-Azzawi et al^5^ showed that 37.8%, 40.6%, and 46.9% (respectively for NCEP/ATP III 2005, IDF 2005 and (2009) revised IDF definition) of Iraqis aged 25-85 at the Baghdad Teaching Hospital were diagnosed with MetS. Additionally, 40.9% (IDF criteria) of people in Erbil, Iraq aged ≥18 year were diagnosed with MetS.^14^ Lastly, 40.6% (42.8% females and 36.5% males) of participants in a Baghdad study of obese adults with different ages have MetS.^15^

A systematic survey of the relevant studies revealed that there is a significant frequency of MetS among college students, which is consistent with these findings.^8^ This is due to the fact that young people who have recently started college are at a crucial age for the creation of good routines and behaviors that fall into routines. For example, first-year college students have a greater propensity to experience a more rapid increase in body mass than the usual adult. The use of tobacco, the drinking excessive amounts of soft drinks, and the consumption of an unhealthy diet are all instances of poor lifestyle choices. It has been demonstrated that the existence of these risk factors, in conjunction with obesity, increases the likelihood of developing MetS in individuals of any age, regardless of their educational or occupational history.^8^

The MetS prevalence could vary between studies according to the MetS cluster used, target population, obesity, and the participant’s lifestyle (irregular meal time, physical inactivity, and increased stress). Also, because there are several methods for diagnosing MetS depending on the standards and thresholds set by the authorities, these factors differ greatly from country to country due to various MetS diagnosis cut-off values and criteria.^16^

The findings of the current study demonstrate that FBG was the most frequent component of MetS (98.3%), followed by WC (87.9%), and finally, HDL-C (85.4%). This result in agreement with the study by Alowfi et al^16^ which report high-FBG and high WC were the most prevalent MetS components in adolescent students in Saudi Arabia.

Various factors, including diet, can cause high blood sugar levels. Excess sugar and carbohydrate consumption increases blood sugar levels after meals because the food is broken down into glucose molecules that enter the bloodstream.

The studies by Mahrous et al^11^ in Egypt and Naghipour et al^1^ in Iran showed that central obesity was the most prevalent component (41.8% and 75.8%, respectively) of MetS among the students. While another study in Colombia reported low HDL C levels as the most frequent MetS component,^17^ and a study by Mbugua et al^18^ found the most prevalent component was elevated triglycerides for university students in Kenya.

In the current study, Age, gender, BMI, physical activity and type of foods are the most important risk factors and lifestyle factors that effect on the incidence of Mets, as shown in (Table 5).

A statistically significant association was observed between the magnitude of MetS and increasing age, ranging from 43.5% in the group of 18-21 year olds to 56.5% for those aged 22-25 years, a result that agreed with rates found by Roos et al.^19^

The study presented here shows that female, as compared to the male participants, had higher MetS (66.9%, 33.1%) respectively, with (OR=2.32, 95% CI=1.4-3.7); a similar finding to an analysis of Pai et al. was noted 2, and agreed with Zafar et al,^20^ who carried out their study among 2982 Indians and found that females (13.8%) had MetS more than male (9.6%). Although the specific cause of such gender differences is unknown, it has been suggested that females are less active than males.^11^

The main risk factors for MetS development include obesity and abdominal obesity (OR=9.46). This result was agreement with the study by B. Damiri et al^21^ who reported MetS prevalence among overweight and obese university Palestinians students was high. Another study by Balgoon et al^12^ shows young female university students in Saudi Arabia with higher BMI values had a higher chance of having MetS, and this increasing BMI also correlates with other MetS components.

Regarding physical activity, participants had a high level of physical activity had a significantly low rate of MetS (OR=0.54, 95% CI=0.48-0.60). Numerous studies have demonstrated that exercise lowers the incidence of MetS.^22^ Increased levels of leisure-time physical activity are linearly related with a decreased risk of MetS, whether in duration or intensity.^23^

In this study, 91.1% of participants had fast food, and 56.6% of them had more than 3 times/week. High consumption of fast food was substantially linked to MetS (OR=2.35, 95% CI=1.42-3.86). Although the students were aware of the risks to their health posed by consuming fast food, their eating patterns did not suggest that they were engaging in behavior that could be detrimental to their health. This was particularly true when the students were interacting with their friends, who tended to consume more fast food than the students themselves did.^24^

Fast food intake has negative consequences on the pandemic of overweight and obesity, fast food eating has been identified as a significant contributor to obesity and rapid weight gain among a number of dietary variables and high risk for metabolic disease.^25^

### Study limitations

The study has a small sample size and was carried out in a single institution. Also different ethnicity (Arabs, Kurds, and Turkmans), and country wide study were not included.

In conclusion, this study shows that the high prevalence of MetS in university students, FBG was the most common component followed by increased WC, and the significant risk factors for MetS were older age, females, high BMI (≥25), low physical-activity, and eating of fast foods. This last result raises the possibility that high FBG may be an important indicator of early pathology linked to the onset of MetS, so adoption of preventive lifestyle modification (like healthy eating behaver and increased physical activity) is recommended to avoid development of MetS among young adult.

Further studies with more studies on this population are needed to help build a comprehensive screening and intervention approach for university students. In addition, investigating the importance and significance of high FBG levels in collage students may have significant benefits for public health, since treatment aiming at decreasing high FBG levels may minimize the future occurrence of MetS and associated clinical disease. Lasty, study MetS prevalence among high school students, and there should be educational programs that encourage healthy eating and provide support for those activities at school.
